# On Sending in Medicines

**Published:** 1811-11

**Authors:** 

**Affiliations:** London


### *To the Editors of the Medical and Physical Journal.*

Gentlemen,

Having long witnessed the prevailing prejudices of the public respecting the mode which is adopted by apothecaries of sending in medicines, I take the liberty of offering two or three observations on the subject. Amongst the middling orders of society, the prevailing cry is, that if an apothecary is consulted, no matter in what case, whether medicines are absolutely necessary or not, he is sure to send in *loads* (as the term is) of medicines; and before a week has elapsed, the patient begins to loath them, either from an idea of their expence, or the frequency of their repetition. Thus the general idea is confirmed, that one half or less of the medicine is *not necessary;* and to strengthen and confirm these prejudices, (whether the cause of them is real or not), the physician *too frequently*, if he is called in unknown to the apothecary, or happens to have an opportunity of seeing the patient alone, broaches the same fascinating doctrine, viz. that so much medicine is absolutely unnecessary; that he believes there is a great deal too much given; and that he is a most decided

 enemy enemy to such practice, and as great a friend for *dieting, rest, &c.* Thus the patient's prejudices are confirmed by the physician: he gains esteem and favour; and his end is answered, as his fee is just the same, whether the invalid takes a larger or a smaller quantity of medicine. I am sorry to observe, that I have lately seen an instance where an apothecary, in an acute disease, had ordered a draught to be given every two hours. When the physician called he deprecated giving the medicine so frequently; desired the stomach might be suffered to rest, *(soothing language)*; and, *cook* like, ordered some soup to be given instead. Thus the patient, in an acute disease, where the apothecary was obliged to call three or four times a day, was suffered to be without medicine for twelve or fourteen hours, merely to humour the patient, gain esteem, and injure the apothecary.

I am perfectly aware that, on many occasions, the apothecary is obliged to send in a proportionate share of medicine, in order to remunerate him for his attendance, he having no other regular *mode of charge*. This is the grand source of evil. If medical men were generally to adopt a plan of charging for their attendance, and include what medicines are *absolutely necessary*, the prejudices of the public must be soon abandoned; and surely such a plan is not unattainable: for instance, if the Company of Apothecaries were to set the example, by adopting a system of that kind—adapting their charges to the different classes of the community they respectively attend—I have no doubt but it would soon be universally followed. Perhaps an objection may be stated, that this kind of payment would be *arbitrary*; but I would ask, is not the present accustomed mode of affixing prices to draughts and mixtures *equally arbitrary*, there being no legalized or enacted *rule* of charge? for it must be observed, that the present existing system is open to the same innovations of arbitrary charge, as the alteration hinted at could ever possibly be subject to: but while matters remain in *statu quo*, and the old plan is adhered to, surely it reflects no great honour or credit on the physician to rob the apothecary of a fair remuneration for his trouble, either by not ordering a fair quantity of medicine, or by sending prescriptions to a chemist, in a family even where a medical man has been in the constant habit of attendance.

OBSERVATOR.

*London, Sept.* 13, 1811.

